# Microfluidic rapid and autonomous analytical device (microRAAD) to detect HIV from whole blood samples†

**DOI:** 10.1039/c9lc00506d

**Published:** 2019-10-09

**Authors:** Elizabeth A. Phillips, Taylor J. Moehling, Karin F. K. Ejendal, Orlando S. Hoilett, Kristin M. Byers, Laud Anthony Basing, Lauren A. Jankowski, Jackson B. Bennett, Li-Kai Lin, Lia A. Stanciu, Jacqueline C. Linnes

**Affiliations:** a Weldon School of Biomedical Engineering, Purdue University, West Lafayette, IN 47907, USA.; b Interdisciplinary Engineering, Purdue University, West Lafayette, IN 47907, USA; c Environmental and Ecological Engineering, Purdue University, West Lafayette, IN 47907, USA; d School of Materials Engineering, Purdue University, West Lafayette, IN 47907, USA

## Abstract

While identifying acute HIV infection is critical to providing prompt treatment to HIV-positive individuals and preventing transmission, existing laboratory-based testing methods are too complex to perform at the point of care. Specifically, molecular techniques can detect HIV RNA within 8–10 days of transmission but require laboratory infrastructure for cold-chain reagent storage and extensive sample preparation performed by trained personnel. Here, we demonstrate our point-of-care microfluidic rapid and autonomous analysis device (microRAAD) that automatically detects HIV RNA from whole blood. Inside microRAAD, we incorporate vitrified amplification reagents, thermally-actuated valves for fluidic control, and a temperature control circuit for low-power heating. Reverse transcription loop-mediated isothermal amplification (RT-LAMP) products are visualized using a lateral flow immunoassay (LFIA), resulting in an assay limit of detection of 100 HIV-1 RNA copies when performed as a standard tube reaction. Even after three weeks of room-temperature reagent storage, microRAAD automatically isolates the virus from whole blood, amplifies HIV-1 RNA, and transports amplification products to the internal LFIA, detecting as few as 3 × 10^5^ HIV-1 viral particles, or 2.3 × 10^7^ virus copies per mL of whole blood, within 90 minutes. This integrated microRAAD is a low-cost and portable platform to enable automated detection of HIV and other pathogens at the point of care.

## Introduction

Despite the effectiveness of antiretroviral therapy (ART) to suppress viral loads and decrease HIV-related mortality, HIV remains a global epidemic. The World Health Organization (WHO) estimates that of the 36.7 million people currently living with HIV worldwide, only 48% are being treated.^1^ Early diagnosis of HIV decreases mortality and morbidity by initiating early patient treatment.^2^ HIV screening is currently performed using commercially-available rapid diagnostic tests (RDT), typically based on lateral flow immunoassay (LFIA) technology that detects HIV antibodies from oral fluid or capillary blood. The low sensitivity during the pre-seroconversion phase of the first four weeks of infection and frequent false negatives require that antibody-detecting RDT results are confirmed by a second or even third laboratory-based assay.^3^ Even the fourth and fifth generation assays that combine antibody and HIV p24 antigen detection in a combo RDT are less sensitive than laboratory-based assays.^4^ However, the delay of diagnosis due to laboratory-based testing significantly impairs an HIV-positive patient's prompt treatment.^5^

Point-of-care (POC) nucleic acid-based diagnostic tests could expedite treatment response for vulnerable and newly infected individuals through early detection of the HIV virus. Reverse transcription polymerase chain reaction (RT-PCR) has been performed in microfluidic-based sample-to-answer devices to amplify HIV RNA spiked into saliva samples. However, the complexity of manufacturing a device to perform both sample preparation and cyclical heating often makes it prohibitively expensive for low-resource settings.^6^ These sensitive detection systems are not cost-effective for early screening and POC testing because they require expensive supporting sample preparation units, cold-chain storage of reagents, off-chip pumps, and trained users.^3,7^ To address these shortcomings, recent efforts have been focused towards developing integrated sample-to-answer nucleic acid analysis devices that can be used by minimally-trained personnel.^8,9^ While these integrated nucleic acid analysis devices minimize user steps and costs, to-date we are unaware of any such devices capable of analyzing viral HIV RNA from whole blood samples. There are a few commercial tools for near-patient detection of HIV including Cepheid Xpert Qual Assay, Alere q HIV-1/2 Detect, and Diagnostics for the Real World's Samba II. Although these tests are able to integrate and automate sample preparation, they all require cost-prohibitive (>$17 000 for the instrument and >$17 for the cartridge) benchtop instruments that need stable electrical power supply or consumable batteries.^10^

Recent advances in technologies for point-of-care molecular detection of HIV include several isothermal nucleic acid amplification techniques that could reduce the complexity and therefore cost of a fully integrated testing device.^11^ One such isothermal amplification method, loop-mediated isothermal amplification (LAMP), provides specific and efficient amplification of target nucleic acids by targeting 8 unique sequences.^12^ The isothermal heating (most efficiently between 65 and 72 °C)^13,14^ of LAMP both lyses many pathogens and robustly amplifies DNA even in the presence of complex sample matrices, further reducing sample processing and instrumentation requirements.^15-17^ To expedite sample preparation steps, such as reverse transcription (RT) of HIV RNA targets to amplifiable DNA, several groups have demonstrated that RT can be performed using the same assay conditions as LAMP.^18-21^ Gurrala *et al*. used RT-LAMP to amplify HIV-1 RNA and produce a pH change that can be measured with their device.^22^ The lack of reagent storage and integrated sample preparation, however, decreases the translatability of this and other RT-LAMP devices.

**Figure 1.**
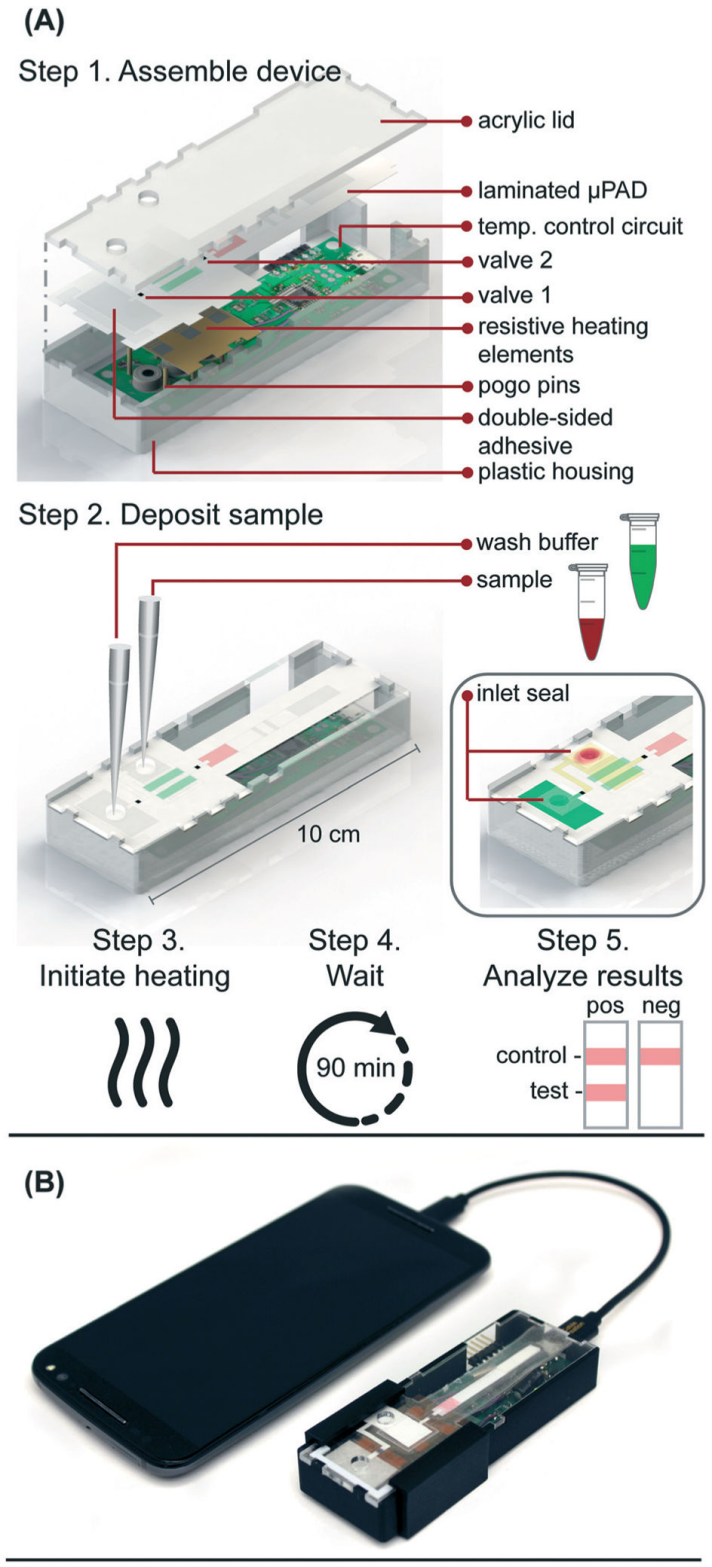
**(A)** Schematic of microRAAD for HIV testing work flow in which user 1) assembles consumable μPAD into plastic housing with reusable resistive heating elements and heating control board, 2) deposits sample and wash buffer into inlets and seals with tape to minimize evaporation, 3) initiates heating by connecting to phone, 4) waits 90 minutes for automated fluid delivery and sample incubation in μPAD, and 5) analyzes results of lateral flow immunoassay. **(B)** Photo of microRAAD connected to phone to power the heating control board.

Here we report a fully-integrated sample-to-answer platform (Fig. 1) that leverages paper membranes' wicking abilities and size discriminating pores to a) isolate HIV viral particles from human blood cells, b) amplify RNA from the viral particles using pre-dried RT-LAMP reagents that target the highly conserved *gag* gene of HIV-1, and c) automatically transport RT-LAMP amplicons to an integrated LFIA for simple, visual interpretation of results within 90 minutes of sample application. This microfluidic rapid and autonomous analysis device (microRAAD) demonstrates the potential for simple and low-cost HIV detection at the point of care.

## Experimental

### Reagents

Reagents necessary for the RT-LAMP assay included six primers (Integrated DNA Technologies, Skokie, IL), Bst 3.0 polymerase (NEB, Ipswich, MA), deoxynucleotide triphosphates (dNTPs) (Agilent Technologies, Santa Clara, CA), isothermal buffer II (NEB, Ipswich, MA), betaine (Millipore Sigma, Burlington, MA), EvaGreen (VWR International, Radnor, PA), ROX (Thermo Fisher Scientific, Waltham, MA), diethyl pyrocarbonate (DEPC) water (Invitrogen, Carlsbad, CA), and human whole blood collected in sodium citrate (Innovative Research, Novi, MI).

Template used in the experiments below included purified genomic RNA from HIV-1 (ATCC, Manassas, VA), non-infectious HIV-1 virus diluted in AccuSpan plasma (AccuSpan Linearity Panel, SeraCare Life Sciences, Milford, MA), purified genomic RNA from dengue virus (DENV) type 1 (BEI resources, Manassas, VA), and purified RNA from chikungunya virus (CHIKV) S-27 (BEI resources, Manassas, VA). *Sph*I and *Pst*I restriction enzymes (NEB, Ipswich, MA) and phosphate buffered saline (PBS) (Thermo Fisher Scientific, Waltham, MA) are additional reagents used.

### Reverse transcription loop-mediated isothermal amplification (RT-LAMP) assay

Purified genomic RNA from HIV-1 or non-infectious HIV-1 virus was used at specified concentrations as the template in preliminary testing and in experiments thereafter. LAMP primers were devised using PrimerExplorer v5 to target the *gag* gene of HIV-1, and loop primers were labeled with 6-carboxyfluorescein (FAM) and biotin for detection *via* commercial LFIA (Ustar Biotechnologies, Hangzhou, China). The primer sequences are provided in Table S1.† To allow for both reverse transcription and amplification of the HIV-1 target, we used Bst 3.0 polymerase, which includes reverse transcriptase capabilities, and the buffers and dyes listed in Table S2† for a 25 μL reaction. 2–4 μL of template (RNA or virus) or negative control (DEPC water or AccuSpan plasma) were added directly to the RT-LAMP master mix prior to heating. A range of temperatures, 58–77 °C, were tested to determine the optimal assay temperature. After heating at indicated temperatures, RT-LAMP amplicons were added to LFIAs for analysis. Hereafter, RT-LAMP was performed at 65 °C for 60 minutes using an Applied Biosystems 7500 Real-Time PCR System (Foster City, CA). The specificity of the target sequence to HIV-1 was verified by BLAST as well as experimentally using 10^5^ copies per reaction of genomic RNA from DENV type 1 and CHIKV S-27. Further, to confirm the identity of the amplified product, the RT-LAMP amplicons were subjected to restriction enzyme digest with *Sph*I and *Pst*I for 1 hour at 37 °C and the digested segments were confirmed *via* 2% agarose gel electrophoresis.

For limit of detection (LOD) experiments, RNA or virus template was prepared by performing 10-fold serial dilutions in either DEPC water (RNA) or AccuSpan plasma (virus). For analysis of RT-LAMP in biological sample matrices, increasing amounts of human whole blood was added into the master mix. Real-time fluorescence data of EvaGreen intercalating dye and ROX reference dye was monitored to confirm the amplification progress. RT-LAMP amplicons were characterized *via* LFIA and confirmed *via* gel electrophoresis using a 2% agarose gel run at 100 V for 60 minutes, stained with ethidium bromide, and imaged using an ultraviolet light gel imaging system (c400, Azure Biosystems, Dublin, CA).

### RT-LAMP reagent drying and storage

Reagents and conditions for drying and storage were modified and adapted from a published protocol.^23^ The RT-LAMP reagents were deposited, dried, and stored at room temperature, eliminating the need for cold-chain storage (−20 °C) and improving the portability of the device. The primer mixture (Table S3†) containing primers, sucrose, glycerol, and Triton X-100 was deposited by hand on 1 cm wide polyethylene terephthalate (PET) film (Apollo, Lake Zurich, IL) in two parallel lines (Fig. S1†) at approximately 1.83 μL cm^−1^. After drying in a sterile biosafety cabinet under continuous air flow for 60 minutes at room temperature, the enzyme mixture containing Bst 3.0 polymerase, sucrose, and dNTPs was deposited directly on top of the dried primers in parallel lines at approximately 1.19 μL cm^−1^ and set out to dry for another 60 minutes. The PET with dried reagents was cut into 1 × 1 cm pieces, corresponding to one 25 μL reaction.

The reagents for the stability studies were packaged after deposition and initial drying and stored in opaque Mylar bags with silica gel desiccant (Uline, Pleasant Prairie, WI) at room temperature for 3 weeks. The dried RT-LAMP reagents were rehydrated with buffer (Table S3†) and virus (positive samples) or DEPC water (negative controls) in tubes or in the polyether sulfone (PES) amplification zone within the integrated device.

### Blood separation and virus capture in paper membranes

As an initial proof of concept of size-based separation, two sizes of fluorescent particles (Bangs Laboratories, Fishers, IN) were used in vertical flow filtration to allow quantification of the membrane capture efficiency. The 0.11 μm diameter Dragon Green (Ex480/Em520 nm) particles represented HIV-1 virus and the 7.32 μm diameter Suncoast Yellow (Ex540/Em600 nm) particles represented red blood cells. The fluorescent particles were diluted in PBS according to manufacturer's instructions. A calibration curve correlating particle concentration to fluorescence was created by serially diluting both particle solutions and measuring dilutions in a SpectraMax M5 microplate reader (Molecular Devices LLC, San Jose, CA) at excitation of 480 nm or 540 nm for the 0.11 μm and 7.32 μm particles, respectively. A 7 mm hole punch was used to cut pieces of blood separation membrane (MF1, GE Healthcare, Chicago, IL) and amplification membrane (0.22 μm PES, Millipore Sigma, Burlington, MA). The cut membranes were sandwiched between two O-rings and placed into a commercial miniprep spin column (Fig. S2†) (Qiagen, Hilden, Germany). The spin column was then placed into a clear 2 mL collection tube.

One hundred fifty (150) μL of either the 0.11 μm or 7.32 μm particles was pipetted into the spin column containing the membrane of interest. The tubes were then centrifuged for 60 seconds at 0.5 rcf and the fluorescence of the eluent was measured and compared to the calibration curve intensities. Fluorescence of unfiltered particles was measured and used as the baseline to calculate the proportion of particles that passed through the membrane.

MF1 and 0.22 μm PES membranes were then used to show size-based capture in a lateral flow format. The 0.22 μm PES (2.5 cm × 1 cm) was overlapped with the MF1 membrane (1 cm × 1 cm) to form the amplification and filtering segments of the integrated device (Fig. S10†). First, a 100 μL solution containing approximately 7 × 10^5^ of 0.11 μm particles, 230 of 7.32 μm particles, and deionized water were mixed to form the particle mixture. Higher concentrations of the 7.32 μm particles oversaturated the fluorescence measurements. Thirty (30) μL of the particle solution was pipetted onto the assembled MF1/PES membrane, followed by a 30 μL PBS wash. The particles in the membranes were immediately imaged at 40× magnification with an inverted Axio Observer Z1 Fluorescent microscope and ZenPro software (Carl Zeiss Microscopy, Thornwood, NY) using a rhodamine dye filter cube for the 7.32 μm particles and an Alexa Fluor 488 dye filter cube for the 0.11 μm particles.

To demonstrate nucleic acid amplification of viral particles after separation from blood cells in the lateral flow format, MF1 and 0.22 μm PES membranes were overlapped as in the particle lateral flow. Next, 1.2 μL of 2.5 × 10^5^ virus copies per μL HIV-1 was mixed with 12 μL of human whole blood and deposited onto the MF1 membrane of the MF1/PES assembly, followed by a 61.8 μL wash of rehydrating mixture (final concentration of 4 × 10^6^ virus copies per mL of reaction volume) (Table S3†). After 1 minute of capillary flow, the PES was removed from the assembly and added into a PCR tube with 23 μL of the enzyme and primer mixtures. The samples and a positive amplification control (reaction without blood or membrane) were amplified for 60 minutes at 65 °C. Amplification was confirmed by placing the PES membranes and control into wells of an agarose gel and performing gel electrophoresis. The remaining solution in the PCR tube that had not saturated the PES membrane was added to a LFIA followed by 40 μL of wash buffer.

### Resistive heating

Resistive heating elements were fabricated by printing JSB40G Nanosilver Ink (Novacentrix, Austin, TX) onto a Kapton substrate (Kapton HN Semi-Clear Amber Film, 5 mm × 125 μm) using a Dimatix Materials Printer DMP-2850 (Fujifilm Dimatix Inc., Santa Clara, CA) with a drop spacing of 35 μm and a platen temperature of 50 °C. Optimization and characterization were performed previously. The printed traces were then cured in an oven at 400 °C for 10 minutes. Individual traces were measured post-curing with a handheld multimeter to determine the average electrical resistance of the heating element. The design in Fig. S3† provided even heating of the amplification zone with an average resistance of 5 Ohms and required an average of 240 mW to reach 65 °C, the temperature necessary for the RT-LAMP assay. The heating elements used to actuate the wax valves also had an average resistance of 5 Ohms and heated to 80 °C using an average of 440 mW. The heat produced by the silver trace dissipates through the Kapton substrate and into the membranes above. The insulation required to maintain steady heating is provided by an acrylic lid and plastic housing pictured in Fig. 1A.

### Temperature control circuit

In the integrated microRAAD, temperature regulation of the resistive heating elements was achieved using a miniaturized, custom-designed electronic device. It was constructed to individually monitor and regulate the temperature of each of three heating zones: one heating zone was dedicated to amplification and two heating zones were dedicated to actuating the wax valves. The temperature control circuit was equipped with a microcontroller (ATMEGA328P-AUR, Microchip Technology, Chandler, AZ), three non-contact infrared (IR) temperature sensors (MLX90614ESF-BAA-000-TU, Melexis Technologies NV, Ypres, Belgium), and three transistor-based current drivers (IRLML6244TRPBF, Infineon Technologies, Neubiberg, Germany) driven by three separate 10 bit digital-to-analog converters (DAC6311, Texas Instruments, Dallas, TX) for precise monitoring and control of current delivered to the resistive heating elements. Six pogo pins (0907-1-15-20-75-14-11-0, Mill-Max Manufacturing Corporation, Oyster Bay, NY), two for each resistive heating element, were used to complete the circuit between the current drivers and the resistive heating elements placed between the circuit board and the microfluidic paper analytical device (μPAD, Fig. 1A). The microcontroller was loaded with an Arduino bootloader (Arduino Pro Mini, 3.3 V, 8 MHz, SparkFun Electronics, Boulder, CO) and programmed using the Arduino development environment (Arduino IDE v.1.8.8) over a universal serial bus (USB) connection through a future technology devices international (FTDI) serial-to-USB interface (FTDI Serial TTL-232 USB Cable, Adafruit Industries, New York City, NY). All electronic components were sourced from Digi-Key Electronics (Thief River Falls, MN). The circuit boards were fabricated by PCBWay (Shenzhen, China) and were assembled in-house using standard soldering equipment.

The microcontroller was programmed to implement a proportional integral differential (PID) algorithm for maintaining temperature of the amplification zones within the user-specified set points (65 °C for the amplification and 80 °C for each wax valve). PID algorithms are popular closed-loop feedback mechanisms due to their simplicity and effectiveness, making them the *de facto* choice for portable, low computing power electronic devices.^24,25^ The microcontroller sampled the temperature of each zone (sampling at 16.2 Hz for the amplification zone and 13.5 Hz for the valves) using the non-contact IR sensors and compared the measured temperature to the user-specified set point in order to determine the error value, *e*(*t*) (the difference between the measured value and the set point as depicted in eqn (1)). The device then computes the proportional, integral, and derivative terms of the PID algorithm. The proportional term represents the difference between the set point and the measured value, multiplied by the proportional gain, *K*_p_ = 0.05. The integral term is the cumulative error and is computed by summing the integral of the current error with the previous errors, multiplied by the integral gain, *K*_i_ = 0.0001. The derivative term represents the change in the error since the last measurement, multiplied by the derivative gain, *K*_d_ = 0.1.

(1) $$u(t)=K_{p}e(t)+K_{i}\int_{0}^{t}e(\tau )d\tau +K_{d}\frac{d}{dt}e(t)$$

The microcontroller then adjusts the current delivered to the resistive heating elements accordingly. The algorithm is designed to achieve accurate temperature regulation within 0.7 °C of the set point, while minimizing overshoot (1 °C). A serial Arduino interface was enabled between the circuit and a computer allowing real-time monitoring of experimental parameters, including temperature of each zone, time elapsed, and power consumption. We verified the temperature with both an infrared camera (FLIR Systems, Wilsonville, OR) and K-type thermocouple measurement of the top and bottom surfaces of the μPAD using a portable temperature data logger (RDXL4SD, OMEGA Engineering, Norwalk, CT).

In the final implementation, the temperature control circuit was powered through a USB port using a USB on-the-go (USB OTG) enabled cellphone (Samsung Galaxy J3 Luna, Android Version 7.0), which removed the need for the computer, increased portability, and ensured fully automated control of the integrated device without the need for user intervention.

### Integrated microRAAD

The microRAAD for HIV detection is composed of the reusable temperature control circuit and silver ink resistive heating elements and a single-use laminated μPAD, all contained in a plastic housing (Fig. 1A). The base of the plastic housing was designed in SolidWorks and 3D printed on a Fortus 380c printer (Stratasys, Eden Prairie, MN). The 0.08″ thick acrylic lid of the housing (model #11G0670A, Shape Products Menomonie, WI) and components of the single-use μPAD (Table S4†) were designed in Adobe Illustrator and cut with a CO_2_ laser (VLS 3.5, Universal Laser Systems, Scottsdale, AZ). Valve strips were prepared by printing 1.25 mm wide lines of solid wax-ink (Black ColorQube ink, Xerox, Norwalk, CT) onto cellulose membranes (Chr1, GE Healthcare, Pittsburgh, PA) using a Xerox ColorQube 8570 (Norwalk, CT). Membranes were then heated for twelve minutes at 85 °C in a table-top oven (VWR, Radnor, PA) and cut into strips with the laser cutter to create closed valve strips.^26^ Commercially available LFIAs were modified for microRAAD by cutting off the sample pad. PET squares were sterilized with 70% ethanol. Once prepared, all components were hand assembled (Table S4 and Fig. S4†) and laminated with pressure sensitive self-seal adhesive (GBC, Lake Zurich, IL) to minimize evaporation during the assay.

Seventy-five (75) μL of prepared RT-LAMP master mix or rehydrating mixture (Table S3†) containing HIV-1 virus (at a final concentration of 4 × 10^6^ virus copies per mL of reaction volume) were loaded into the sample inlet of the μPAD and sealed with a 1 × 1 cm square of self-seal prior to assembling the μPAD into the plastic housing with the temperature control circuit. This method was used to minimize contamination between experiments, however, we also verified that the sample can be added to the sample inlet once the μPAD is assembled in the plastic housing, which is how we anticipate the device would be used at the point of care.

The assembled microRAAD was then loaded with wash buffer and subjected to localized heating of the amplification zone and valves. When testing whole blood samples spiked with HIV-1 virus, 1.2 μL of 2.5 × 10^5^ virus copies per μL HIV-1 was mixed with 12 μL of human whole blood and loaded into the sample inlet, followed by a 61.8 μL addition of rehydrating mixture (final concentration of 4 × 10^6^ virus copies per mL of reaction volume). The loaded μPAD was then adhered to the acrylic lid with double-sided adhesive at the wash inlet. Resistive heating elements were adhered to the backside of the μPAD, aligned with the two valves and amplification zone, and faced such that the silver traces would contact the pogo pins of the temperature control circuit inside the plastic housing. Two plastic brackets were slid over the acrylic lid and plastic housing to ensure proper contact within microRAAD. Finally, 130 μL of green food coloring solution (for visualization of flow) were added to the wash inlet and sealed. Heating was initiated *via* the serial interface between the computer and the temperature control circuit: 1) 65 °C for the middle resistive heating element (amplification) for 60 minutes and 2) 80 °C for the outer resistive heating elements (valves) for 2 minutes. After 30 minutes of development (1.5 hours after initiating the heating), the LFIA was imaged using a desktop scanner for analysis (Epson, Suwa, Japan).

### LFIA quantification and statistical analysis

All RT-LAMP amplicons were characterized *via* LFIA. The LFIAs were scanned at least 30 minutes after initial sample addition using an Epson V850 Pro Scanner. The test band was quantified using a custom MATLAB script that averages the grey-scale pixel intensity of the test band and subtracts the average background pixel intensity 25 pixels below the test band.^27^ To determine the limit of detection, a one-way ANOVA *post hoc* Dunnett's was performed with multiple comparisons of the LFIA test bands of each concentration against the test bands of no template negative controls with a 95% confidence interval. A Student's unpaired, two-sided *t*-test with a 95% confidence interval was used when comparing the negative control and positive samples during the initial testing of 21 day dried reagents and to determine significance between control and positive samples detected in the integrated device.

## Results and discussion

### RT-LAMP assay design and optimization

Several published HIV LAMP primer sets yielded slow amplification in our hands which we attribute to differences in reverse transcriptase and polymerase activity and the HIV subtype used to design the primers.^18-20^ Moreover, while some primers produced amplicons easily observed by real-time fluorescence and gel electrophoresis, they produced poor LFIA signals possibly due to steric hindrance of the labeled primers at the test band. Therefore, we designed novel primers (sequences provided in Table S1†) to specifically target a 201 base pair sequence in the *gag* gene in HIV-1 subtype B. There is one copy of the *gag* gene in the HIV-1 genome and two copies of the genome per viral particle.^28^ Optimized assay conditions are shown in Table S2† and provided the greatest amplification time difference between positive and negative samples, as observed by real-time fluorescence (data not shown). The optimal RT-LAMP assay temperature for these primers is 65 °C as this temperature yielded the strongest test band intensity on LFIAs illustrated in Fig. S5.† To assess specificity of the RT-LAMP assay, primers were also tested with RNA from DENV and CHIKV. As seen in Fig. S6,† there was no cross-reactivity of the HIV LAMP primers with these other RNA-based viral pathogens. Further confirmation of specificity was provided by performing enzymatic digestions of the amplified product with either *Sph*I or *Pst*I. As predicted by LAMP restriction digest fragment analysis,^29^ digestion with either of these enzymes resulted in smaller fragments compared to the undigested product, with the *Pst*I digested product collapsing to the shortest fragment seen on the agarose gel in Fig. S7.† Taken together, these experiments suggest that the designed primer set targets the intended region in the *gag* gene and that this target amplification is specific to HIV-1.

### RT-LAMP assay performance

**Figure 2.**
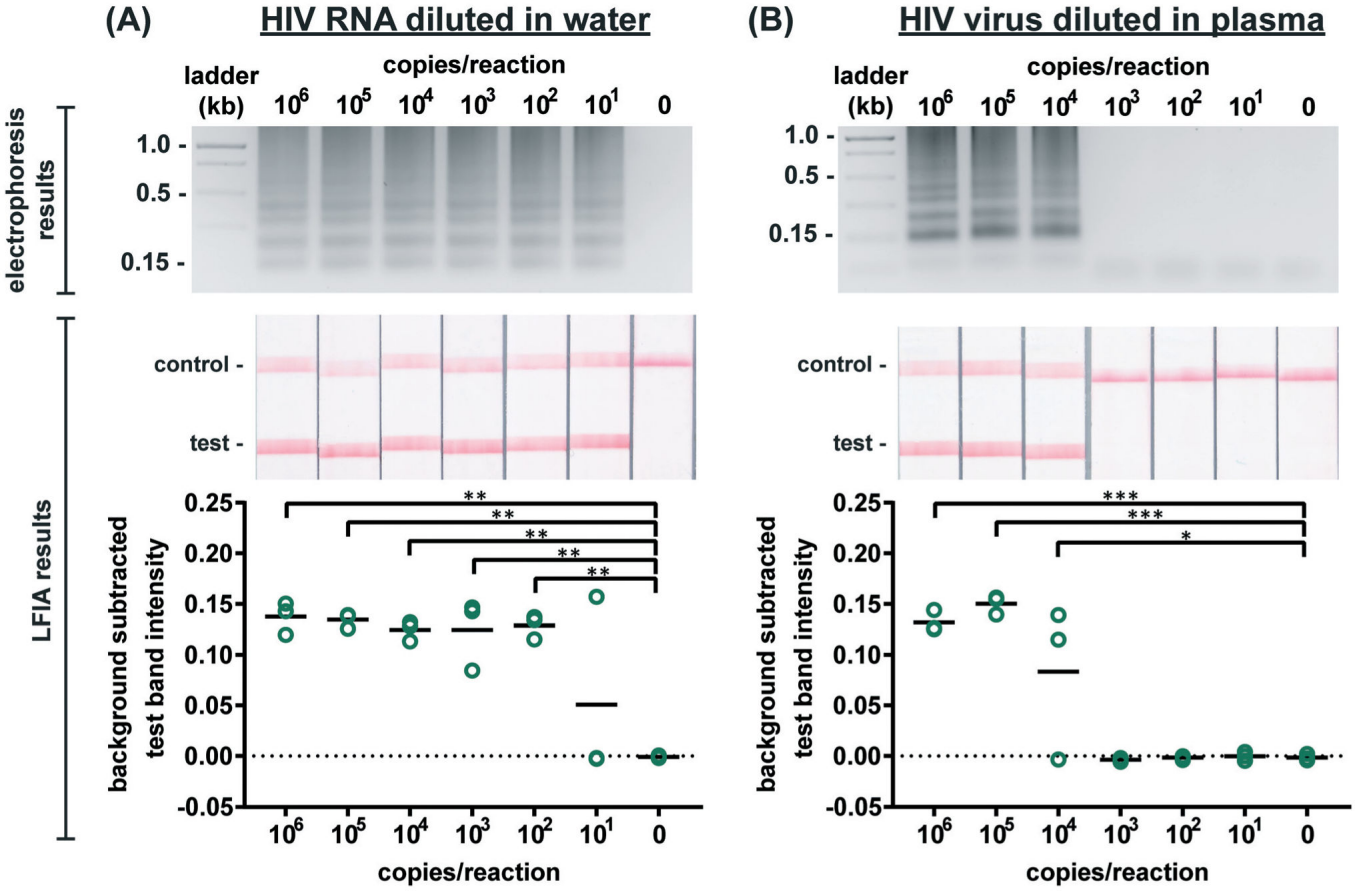
Detection of HIV RNA and virus amplified by RT-LAMP. Electrophoresis gels verifying amplification (top, contrast increased for visualization), LFIA test results (middle), and LFIA test line quantification (bottom). **(A)** Labeled RT-LAMP amplification products are visually detectable from as few as 10 copies of HIV RNA diluted in water. **(B)** Labeled RT-LAMP amplification products are visually detectable from as few as 10,000 HIV viral particles when reactions contain 16% serum. n=3, replicates indicated by each circle; *** indicates p ≤ 0.001; ** indicates p ≤ 0.01; * indicates p ≤ 0.05.

We first evaluated the newly designed RT-LAMP assay using purified HIV-1 RNA at concentrations ranging from 10^1^–10^6^ RNA copies per reaction (*n* = 3). As expected, the real-time fluorescence data displayed faster amplification for samples with higher initial concentrations of RNA (data not shown). Agarose gel electrophoresis confirmed amplification of the RNA after the 60 minute assay (Fig. 2A). The RT-LAMP amplicons were also added to LFIAs and the test band intensity was quantified to determine the LOD. While 10^1^ copies of RNA was detectable in 1 of 3 replicates, statistically significant differences between the test band intensity of the negative control (0) compared to 10^2^–10^6^ RNA copies per reaction (*p*-value < 0.01) (Fig. 2A) demonstrate an assay LOD of 100 copies of HIV-1 RNA per reaction. Other groups have reported a comparable LOD, ranging from 30–250 RNA copies per reaction for their HIV RT-LAMP assay.^18,20,30^

Next, we conducted the RT-LAMP assay with whole HIV-1 virus to ensure sufficient viral lysis at the 65 °C assay temperature. Viral lysis is necessary to release the RNA for amplification. Since the osmotic pressure gradient would prematurely lyse the virus, amplification of virus diluted in water was not tested during characterization studies, as was done with the RNA. Instead, we performed RT-LAMP of HIV-1 virus spiked into reactions containing 16% plasma at concentrations of 10^1^–10^6^ virus copies per reaction (*n* = 3). We used AccuSpan plasma, because the virus stock was supplied in this solution. LFIA analysis showed a statistically significant difference between the test band intensity of 10^4^, 10^5^, and 10^6^ virus copies per reaction compared to the negative control (0) (*p*-value < 0.05 and *p*-value < 0.001, respectively) (Fig. 2B) and the agarose gel supported this conclusion. Therefore, the limit of detection of the assay with HIV-1 virus in 16% plasma is 10^4^ virus copies per reaction. While this LOD is 100× higher than purified RNA in water, when RNA is spiked into 16% plasma, we observed no amplification (data not shown). We suspect that the RNA within viral particles is partially protected from inhibitory factors in plasma until the factors are deactivated and the virus is thermally lysed, but further investigation is necessary to delineate the cause of the loss in sensitivity. Still, this is the first demonstration to our knowledge of an HIV RT-LAMP assay without separate RNA extraction and purification, vastly simplifying the sample preparation required for detection of viruses in complex matrices.

We then evaluated the robustness of the RT-LAMP assay in the more complex matrix of human whole blood. HIV-1 virus spiked into RT-LAMP reactions at a concentration of 10^5^ virus copies per reaction with increasing percentages of whole blood (0–30%) was detectable *via* gel electrophoresis in up to 15% whole blood whereas LFIA detected HIV-1 in up to 20% whole blood (Fig. S8†). In agreement with our observations, other groups have also noticed that LFIA visualization can be more sensitive than gel electrophoresis.^31^ In the integrated microRAAD device, a blood separation membrane, MF1, captures the red and white blood cells, allowing the virus to flow into the amplification zone. Therefore, while whole blood is not intended to be present in the amplification zone, our results indicate an assay tolerance up to 20% in case of poor MF1 capture efficiency or blood cell hemolysis.

### RT-LAMP assay modified for ambient temperature storage

Given that RT-LAMP assays currently require cold-chain storage, we set out to develop a reagent drying method to enable ambient-temperature stable reagents. Others have used LAMP reagent vitrification in PCR tubes to enable ambient-temperature storage,^23^ however, we were concerned that the requirement to separate the enzyme and primers might result in diffusion-limited amplification in the porous membrane of our μPAD. Therefore, prior to experimentation, we calculated the molecular diffusivity of reconstituted reagents into the PES amplification zone using the Stokes–Einstein equation^32^ and Renkin equation.^33,34^ The hydrodynamic radius, *α*, was calculated for the largest molecule in both the primer and enzyme mixture, which was the FIP primer and Bst 3.0 polymerase, respectively. Using eqn (2), we then calculated the bulk diffusivity, *D*, for each molecule where *R* is the gas constant, *T* is temperature, *η* is viscosity, and *N*_A_ is Avogadro's number.

(2) $$D=\frac{RT}{6\pi \eta \alpha N_{A}}$$

Since the diffusion occurs in a porous membrane, we then calculated pore diffusivity, *D*_m_, using eqn (3).

(3) $$D_{m}=DK(\alpha ∕r)\omega_{r}(\alpha ∕r)$$

where *r* is the pore radius, *K*(*α/r*), the partition coefficient, is equal to (1 – *α/r*),^2^ and the hydrodynamic drag, ω_r_(*α/r*), is equal to [1 – 2.1(*α/r*) + 2.09(*α/r*)^3^ – 0.95(*α/r*)^5^]. The predicted pore diffusivity indicates that the time for the reconstituted reagents to diffuse 1 mm through porous PES membrane is approximately 10 minutes. We decided upon layered parallel lines to minimize the distance which the reconstituted reagents would have to diffuse to ensure proper mixing for subsequent amplification.

**Figure 3.**
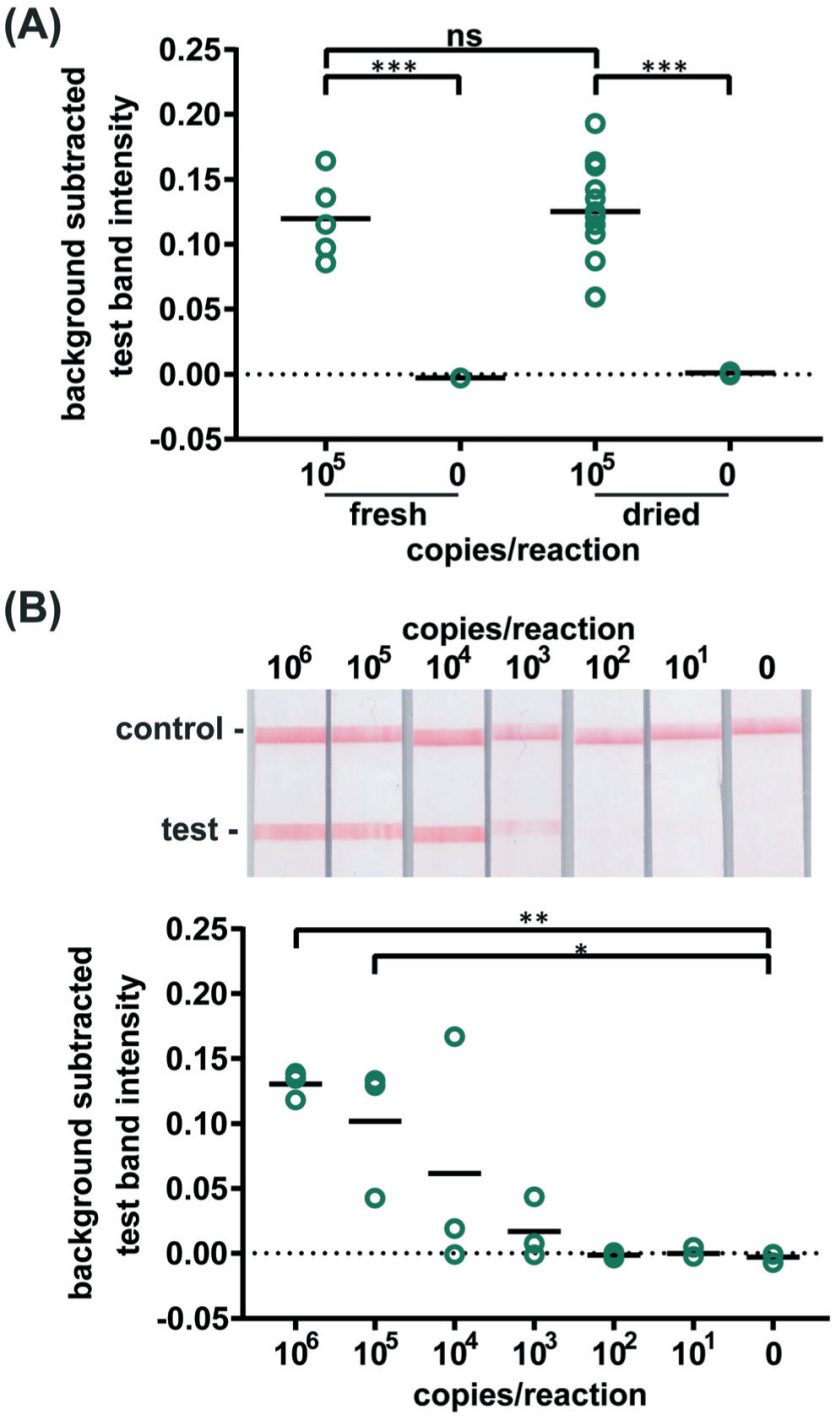
Detection of HIV virus amplified by dried RT-LAMP reagents. **(A)** There is no significant difference in test line intensity of labeled amplification products detected on LFIAs after amplification with fresh RT-LAMP reagents as with reagents dried for 21 days. n=5 (fresh), n=13 (dried), circles indicate replicates; *** indicates p ≤ 0.001 **(B)** Labeled RT-LAMP amplification products are visually detectable from as few as 1,000 HIV virus particles when reactions contain 16% serum. Electrophoresis gels verifying amplification (top, contrast increased for visualization), LFIA test results (middle), and LFIA test line quantification (bottom). n=3, circles indicate replicates; ** indicates p ≤ 0.01; * indicates p ≤ 0.05.

Confident that these assays could work in principle, we experimentally evaluated the robustness of the assay using 21 day dried RT-LAMP reagents. We reconstituted the dried RT-LAMP reagents with rehydrating mixture and HIV-1 at a concentration of 10^5^ virus copies per reaction in water. Positive and negative control reactions using freshly prepared reagents were heated simultaneously. After the 60 minute amplification, samples and controls were analyzed *via* LFIA (*n* = 6, Fig. 3A). The LFIA test band intensity of positive samples using 21 day dried reagents was not statistically significantly different than that of the test band of the freshly prepared positive controls, indicating that the drying process did not damage enzyme or primer activity. As expected, LFIA results of positive samples were statistically differentiable from the negative samples for both the dried and fresh reagent groups (*p*-value < 0.001) (Fig. 3A).

To compare the amplification efficiency of dried and freshly prepared reagents, the LOD of HIV-1 in 16% plasma was determined using 21 day dried RT-LAMP reagents (*n* = 3). There was a statistically significant difference between the test band intensity of the 10^5^ and 10^6^ virus copies per reaction compared to the negative control (0) (*p*-value < 0.05 and *p*-value < 0.01, respectively) (Fig. 3B). While not significant, 10^4^ and 10^3^ copies of virus did amplify in some cases; two of three repeats for 10^4^ and one of three repeats for 10^3^ virus copies per reaction. There is a slight loss in sensitivity when using the dried reagents (LOD of 10^5^ *versus* 10^4^ virus copies per reaction), however, the LOD can likely be improved with further assay optimization such as RT and polymerase enzyme selection and primer design. Another study reported a loss of reaction efficiency using lyophilized HIV RT-LAMP reagents stored at ambient temperature for several hours when compared to freshly prepared controls.^21^ However, Hayashida and colleagues established that vitrified, not lyophilized, LAMP reagents designed for DNA targets have the same sensitivity as freshly prepared reagents after seven months of storage at room temperature.^23^ These findings in combination with our preliminary evaluation of limited HIV RT-LAMP reagents stored for five months, shown in Fig. S9,† give us reason to believe that we can increase the storage time of the HIV RT-LAMP reagents at room temperature far beyond 21 days.

### Blood separation and virus capture in paper membranes

As our RT-LAMP assay was shown to be robust in up to 20% whole blood, we required the removal of the majority of blood cells that would inhibit the reaction. We developed a simple quantitative method to experimentally test the size-based capture of blood cells and virus in membranes. As seen in Table 1, the 7.32 μm particles, representative of blood cells, were captured in the MF1 membrane at an efficiency of 98.6% while 30% of the 0.11 μm particles, representative of the virus, were captured by MF1 (*n* = 3). This implies that MF1 can be used for size-based separation of blood cells from the virus, although some virus will remain in the MF1 membrane, thereby reducing detection sensitivity. Further, 47.6% of the 0.11 μm particles were trapped in the PES membrane (Table 1, *n* = 3), indicating that the PES could localize nearly half of the smaller particles within the amplification zone. Despite the PES membrane having a reported 0.22 μm pore diameter, we suspect that a fraction of smaller diameter particles were trapped in the PES and the MF1 membranes due to a combination of properties. Because of the membrane heterogeneity, a portion of the pores may be smaller than the nominal pore size, allowing particles to be physically trapped. Furthermore, the tortuosity of the membranes may prevent particle migration through the membrane.^35^ Lastly, the proprietary surface chemistries of both membranes may create a slight charge-based attraction that causes particles to adhere to the membranes. Our experimental results indicate a future design that could leverage these factors; extending the PES membrane could enable a filtration and localization of the virus for more sensitive detection from higher sample volumes.

After quantifying the particle separation in columns, we verified successful particle capture in a lateral format by imaging fluorescent particles applied to an MF1/PES membrane assembly, depicted in Fig. S10.† Fig. S11† demonstrates that some of the 0.11 μm particles were trapped in the MF1 membrane, but the majority were dispersed throughout the PES membrane (*n* = 3), aligning with the vertical flow experiment results and indicating that the virus can be separated from the blood cells and localized to the PES amplification zone.

After the characterization with particles, we confirmed that blood cells would be trapped in the MF1 membrane while the virus would flow into the PES membrane for subsequent amplification. We spiked HIV-1 virus into human whole blood and added the mixture onto the MF1 membrane which overlapped with the PES membrane and chased the sample with rehydrating mixture. After removing the PES from the MF1/PES assembly and amplifying the trapped virus in the PES membrane, the amplicons were analyzed *via* LFIA. As depicted in Fig. S12,† the test band intensity is strong, implying that the virus is indeed dispersed throughout the PES (*n* = 3) yet accessible for amplification. Interestingly, when HIV-1 virus diluted in blood was pre-mixed with the rehydrating buffer prior to adding the combined solution to the assembly, amplification was inconsistent and sometimes completely inhibited because more red blood cells seemingly migrated to the PES (Fig. S12†). Our successful membrane amplification results are consistent with previous findings that have also shown that LAMP and other isothermal amplification methods can be performed within the PES membrane.^36^ MF1 membranes inhibited the amplification assay and products extracted from the MF1 were not visible in either the agarose gel or LFIA (data not shown).

### Integration of microRAAD

**Figure 4.**
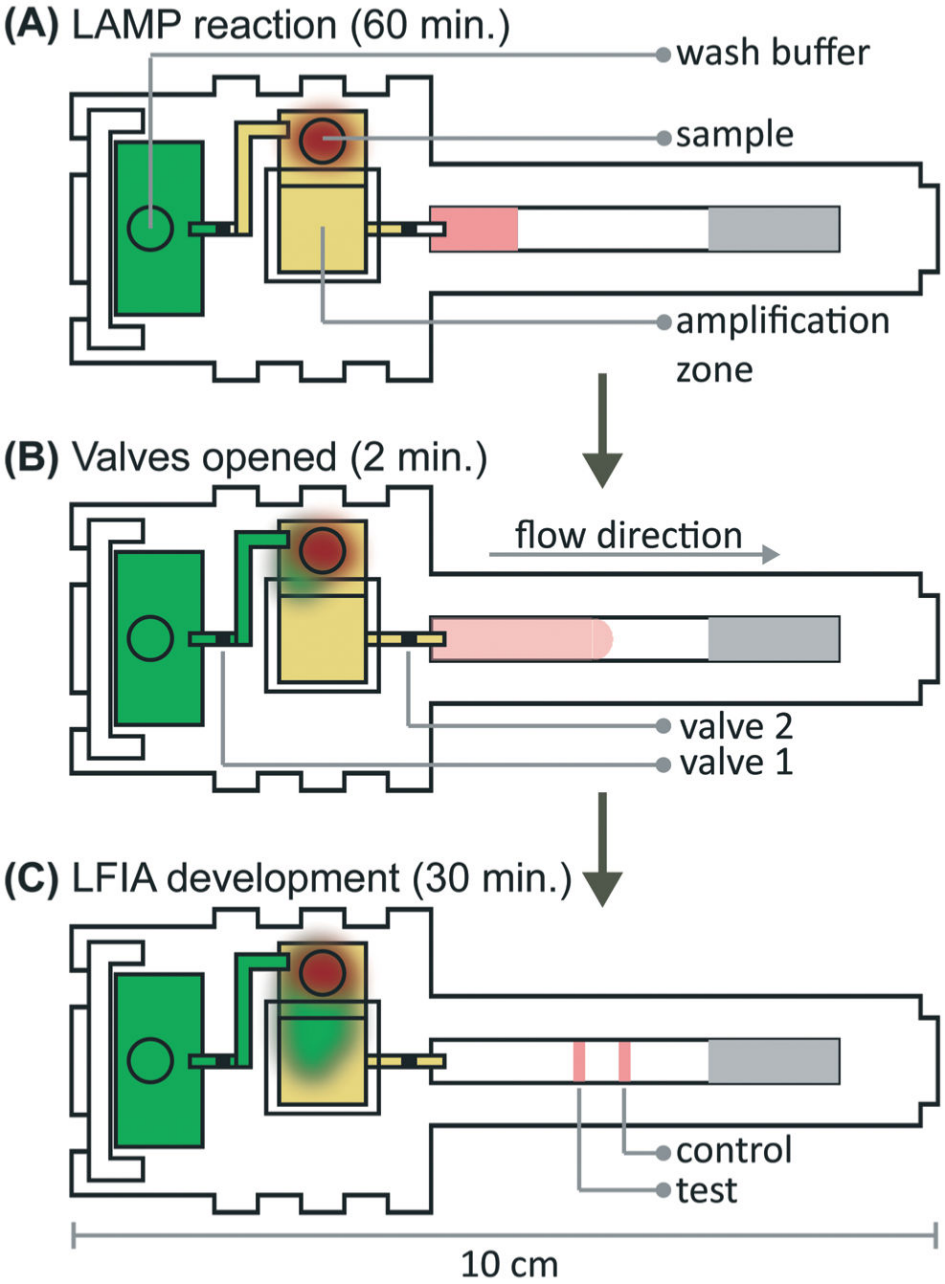
Detection of HIV virus from spiked blood on microRAAD with reagents dried for 21 days. **(A)** Representative μPADs imaged 90 minutes after blood with and without HIV virus deposited into microRAAD’s sample inlets. After capillary migration of HIV to RT-LAMP zone, the RT-LAMP zone and valves are automatically heated, releasing solution to LFIA for detection. As few as 105 HIV virus copies in **(B)** rehydrating mix alone or **(C)** rehydrating mix with 12μL of blood are detectable by microRAAD prepared with RT-LAMP reagents dried for 21 days. n=4 (B) and n=3 (C), replicates indicated by each circle; ** indicates p ≤ 0.05 and ** indicates p ≤ 0.01.

We verified flow in microRAAD with dyed solutions deposited into the sample and wash inlets and then heated the amplification zone and valves. This initial testing indicated 130 μL of wash buffer and 75 μL of sample were required. With too little volume, there was an insufficient pressure gradient to drive flow past the opened valves, while too much volume caused the fluid to prematurely leak past the valves. The wash solution is necessary to sufficiently drive the RT-LAMP amplicons from the PES amplification zone into the LFIA for detection. Initial fluidic designs transported the wash solution directly to the PES, however, given the PES's small pore size and slow wetting, this caused frequent “short-circuiting” of the wash to the LFIA. By directing the wash through the MF1, rather than to the PES directly, we ensured that amplicons in the PES migrated to the LFIA before the wash (Fig. 4 and ESI† Video). Moreover, we verified that no capillary leaks occurred between the membranes and lamination by confirming the flow rates of sealed and unsealed membranes were equivalent (data not shown).

During heating, we observed that the amplification zone reached 65 °C within seconds of initiation and remained at 65 ± 2 °C throughout the 60 minute heating period which is adequate for efficient amplification. In separate experiments, we determined that even low concentrations of template can amplify at temperatures between 62 °C and 71 °C (Fig. S14†). The temperature control circuit automatically terminated the amplification zone heating and initiated simultaneous heating of the wax valves. A 1.25 mm width valve was experimentally verified to sufficiently constrain the wash buffer from the amplification zone; thinner valves leaked wash buffer, diluting the amplicons and potentially decreasing assay sensitivity. We have previously reported that only 41 °C is required to open wax-ink valves prepared in chromatography paper,^26^ however, here we subjected the valves to 80 °C to accelerate their opening. Upon initiation of valve heating, the green wash buffer flowed past valve 1 to the MF1 and the heated sample flowed past valve 2 into the LFIA portion of the μPAD. Within 5–10 minutes of the valves opening, test and control bands were consistently observed on the LFIAs and quantification, in Fig. S13,† revealed strong test band intensities. We found that both a laptop and a cellphone with USB OTG provided sufficient current to power the temperature control circuit for the duration of the assay and yielded comparable results (Fig. 1B).

**Fig. 5:**
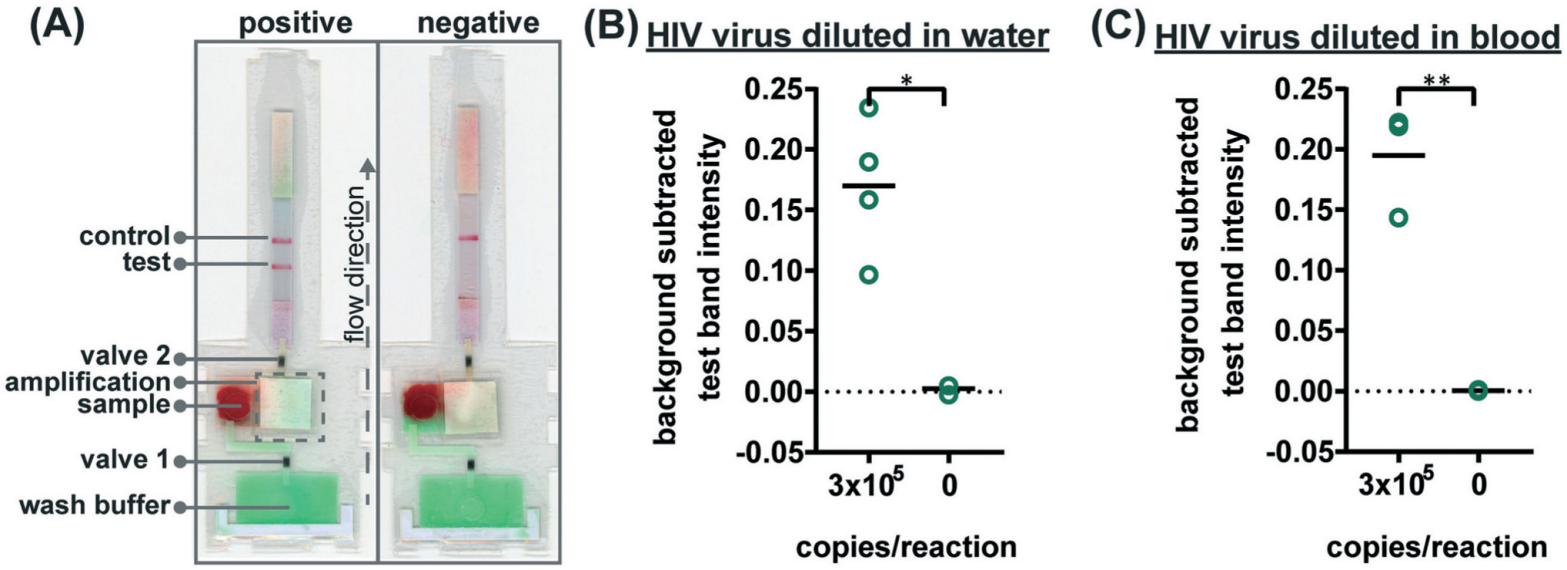
Detection of HIV virus diluted in whole blood on microRAAD with reagents dried for 21 days. (A) Representative μPADs imaged 90 minutes after blood (with and without HIV virus) deposited into microRAAD's sample inlet. After capillary migration of HIV from sample inlet to amplification zone and subsequent heating, the valves are automatically heated, releasing solution to LFIA for detection. As few as 3 × 10^5^ HIV (B) osmotically lysed virus copies in rehydrating mix alone or (C) intact virus in rehydrating mix with 12 μL of blood are detectable by microRAAD prepared with RT-LAMP reagents dried for 21 days. *n* = 4 (B) and *n* = 3 (C), replicates indicated by each circle; ** indicates *p* ≤ 0.05 and ** indicates *p* ≤ 0.01.

To verify the amplification functionality of microRAAD, we initiated the automated detection using 21 day dried RT-LAMP reagents and rehydrating buffer containing HIV-1 virus osmotically lysed in water. Samples containing as few as 3 × 10^5^ virus copies per reaction resulted in unequivocally positive test bands and samples containing no template (0) yielded negative test results (*p*-value < 0.05) (Fig. 5B). The test band intensity at a concentration of 3 × 10^5^ virus copies per reaction using the dried reagents in microRAAD was comparable to the test band intensity of the same concentration in a tube reaction with the dried reagents (Fig. 5B and 3A).

Finally, we performed the detection in the integrated microRAAD using 21 day dried amplification reagents and HIV-1 virus in whole blood. As expected and seen in Fig. 5A, the red blood cells remained in the MF1 directly below the sample inlet while the remaining plasma and buffer solution with virus migrated to the PES for amplification. Following amplification, we visually observed positive test bands on the LFIAs within 5–10 minutes after valves opened. There was a statistically significant difference between the test band intensity of the 3 × 10^5^ virus copies per reaction compared to the negative control (0) (*p*-value < 0.01) (Fig. 5A and C). Notably, the sensitivity using microRAAD for HIV-1 viral detection in blood using dried reagents is comparable to the sensitivity of standard tube reactions with similar conditions (Fig. 5C and 3B). Given that the PES membrane absorbs about 20 μL, which is a comparable volume to tube reactions, we expect that diffusion limitations were minimal, further confirmed by our calculations of diffusivity within the PES membrane. Previous groups have similarly reported only 5 to 10-fold reductions in sensitivity when translating manual assays into automated sample-to-answer devices.^8,37,38^ Liu *et al*. designed a device to detect viral RNA from oral fluid samples in real time down to 12.5 virus copies per reaction, however, viral lysis is required before sample addition and is followed by four more manual steps prior to initiation of the RT-LAMP assay.^39^ Damhorst *et al*. developed a microfluidic chip for blood cell lysis and modified a smartphone for real-time detection of HIV-1 virus with an LOD of 1.7 × 10^4^ virus copies per reaction.^21^ However, the user is required to transfer the lysed blood and freshly prepared RT-LAMP reagents to the reaction chamber for amplification.^21^ Even though this platform is 10-fold more sensitive than microRAAD, we believe that the full automation of microRAAD, which reduces sample handling and exposure to bloodborne pathogens, makes it an advantageous system for rapid HIV testing at the POC.

Our initial studies of this integrated sample-to-answer device demonstrate its potential to provide simple, affordable, and rapid detection of HIV from blood samples at the point of care. The consumable components of microRAAD (membranes, LFIA, adhesive, reagents) cost only $2.23 per assay (Table S4†) while the reusable components (temperature control circuit and housing) are $70.08 and expected to decrease with increased production (Table S5†). The price of components is comparable to other rapid HIV tests developed for resource-limited settings and will decrease as we scale-up the manufacturing of the device.^40^ While low component cost does not guarantee a low price point for consumers, it remains a critical feature of research and development that we considered.^41^ Even though microRAAD has many advantages over comparable diagnostic tools, there remain some limitations. The sensitivity of this integrated prototype is 3 × 10^5^ virus copies per reaction, or 2.3 × 10^7^ virus copies per mL of whole blood, which falls at the high end of the clinical range, 10^7^ virus copies per mL at peak infection at day 17.^42^ This reduced sensitivity may be due to microRAAD's performance of thermal lysis within the device that bypasses nucleic acid purification and concentration steps. However, the automation of virus analysis significantly streamlines testing and potentially reduces user error. We expect that additional improvements in primer design, RT and polymerase enzyme selection, and the addition of virus concentration from larger volumes of sample could further improve the device's sensitivity and enhance clinical utility. Specifically, the incorporation of a smaller pore-sized membrane could enable size-based capture of virus and isolation from inhibiting blood components. Additionally, including an internal amplification control into microRAAD could differentiate negative from invalid results.^37^ Incorporating these improvements along with extended device storage and usability studies will enable clinically relevant detection and early diagnosis of HIV at the POC.

## Conclusions

We have demonstrated microRAAD, an autonomous and fully integrated sample-to-answer device, for the specific detection of HIV-1 virus from human whole blood. After sample addition, the LFIA can be visualized within 90 minutes. Moreover, the user is required to perform only four steps to initiate the testing: load sample and rehydrating mixture, add wash buffer, seal the inlets with adhesive, and initiate the temperature control circuit by connecting a power source such as a computer, cellphone, or portable battery. One of the most noteworthy aspects of microRAAD is the complete automation from blood-in to results-out; minimizing sample preparation and time-critical steps by the user. Furthermore, we have developed a novel RT-LAMP assay and demonstrated that reagents can be dried and stored at room temperature for three weeks before use in the integrated device. The ability to dry reagents eliminates the need for cold chain storage and increases the usability and portability of the device, especially in resource-limited settings. The sensitivity of this integrated prototype is 3 × 10^5^ virus copies per reaction, or 2.3 × 10^7^ virus copies per mL of whole blood, which is comparable to clinically reported HIV-1 concentration at the peak of infection.^42^

MicroRAAD has the potential to serve as a platform for detection of other pathogens. By modifying the LAMP primers to target a new gene of interest and adjusting sample preparation depending on the pathogen target and sample matrix, this platform could be used for other viruses (*e.g*. DENV, CHIKV) and even bacteria (*e.g. Escherichia coli, Vibrio cholerae,* or *Bordetella pertussis*) and parasitic (*e.g. Plasmodium falciparum*) pathogens. This rapid, integrated, and automated device lends itself to use in low-resource areas where clinics and laboratories are scarce and gold-standard testing can take up to one week.^43^ Moreover, microRAAD requires only $2.23 worth of consumable components, making it an affordable detection tool. MicroRAAD combines robust and selective molecular techniques with elegant capillary fluidics and resilient heating controls into a single, portable platform for rapid pathogen detection at the POC.

## Supplementary Material

ESI

ESI video

## Figures and Tables

**Table 1:** Efficiency of membrane capture of fluorescent particles *n* = 3

Particle size	Membrane	Fluorescence (RFU)	Capture efficiency
**0.11 μm**	None	4007.6 ± 165.0	**0%**
	MF1	2810.9 ± 193.0	**30.0%**
	0.22 μm PES	1986.8 ± 103.2	**47.6%**
**7.32 μm**	None	275.1 ± 12.2	**0%**
	MF1	2.3 ± 1.3	**98.6%**
	0.22 μm PES	0.9 ± 0.2	**81.9%**
