# Supplementary material for: Microfluidic rapid and autonomous analytical device (microRAAD) to detect HIV from whole blood samples
Source: Lab Chip. Author manuscript; Available in PMC 2020 Jul 27. (PMC7384476; doi:10.1039/c9lc00506d)
Supplement: ESI [file NIHMS1603882-supplement-ESI.pdf]

Electronic Supplementary Material (ESI) for Lab on a Chip.
This journal is © The Royal Society of Chemistry 2019

Electronic Supplementary Information

Microfluidic Rapid and Autonomous Analytical Device (microRAAD) to Detect HIV from Whole Blood Samples

Elizabeth A. Phillips,[a†] Taylor J. Moehling,[a†] Karin F.K. Ejendal,[a] Orlando S. Hoilett,[a] Kristin M. Byers,[a] Laud Anthony Basing,[a] Lauren A. Jankowski,[b] Jackson B. Bennett,[c] Li-Kai Lin,[d] Lia A. Stanciu,[d] and Jacqueline C. Linnes[a*]

[a]Weldon School of Biomedical Engineering, Purdue University, West Lafayette, IN 47907

[b]Interdisciplinary Engineering, Purdue University, West Lafayette, IN 47907

[c]Environmental and Ecological Engineering, Purdue University, West Lafayette, IN 47907

[d]School of Materials Engineering, Purdue University, West Lafayette, IN 47907

[†] Indicates equal contribution of this work

Email: jlinnes@purdue.edu (J.C. Linnes)

Table S1. Nucleotide sequences of LAMP primers that target the *gag* gene.

Primer	Sequence (5’ – 3’)
B3	AGTTCCTGCTATGTCACTTC
F3	TCAGCATTATCAGAAGGAGC
BIP	ATGAGGAAGCTGCAGAATGGGCCCTTGGTTCTCTCATCTG
FIP	GGTCTCTTTTAACATTTGCATGGCTTTAAACACCATGCTAAACACA
LB	AGTGCATGCAGGGCCTATTG
LF	TGCTTGATGTCCCCCCAC
LB-FITC	/56-FAM/AGTGCATGCAGGGCCTATTG
LF-Biotin	/5-Biosg/TGCTTGATGTCCCCCCAC

Table S2. RT-LAMP master mix used for amplification of HIV.

**Reagent**	**Concentration**
Isothermal Buffer II	1.0X
dNTPs	1.5 mM
Betaine	200 mM
F3 Primer	0.2 µM
B3 Primer	0.2 µM
FIP Primer	1.6 µM
BIP Primer	1.6 µM
LB Primer	0.8 µM
LF Primer	0.8 µM
EvaGreen Dye	0.2X
ROX Reference Dye	1.0X
Bst 3.0 Polymerase	4 U
Sucrose	165 mM
Glycerol	0.28%
Triton X-100	0.007%
Sample	2-4 µL
DEPC H_2O	Fill to 25 µL

Table S3. HIV RT-LAMP mixtures for reagent drying.

Primer Mixture	Enzyme Mixture	Rehydrating Mixture
44.8 mM Sucrose	120 mM Sucrose	1X Isothermal Buffer II
0.007% Triton X-100	1.5 mM dNTPs	200 mM Betaine
0.28% Glycerol	4 U Bst 3.0 Polymerase	
0.2 µM F3/B3		
1.6 µM FIP/BIP		
0.8 µM LF/LB		

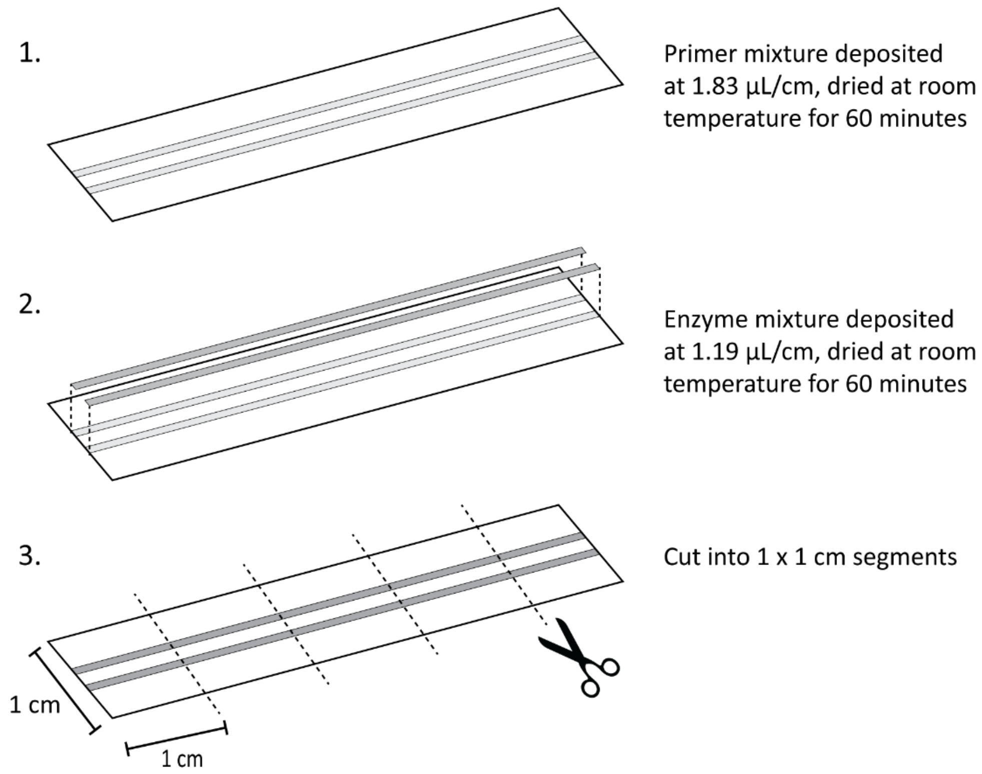

Figure S1. HIV RT-LAMP reagent drying setup. The primer mixture is deposited onto PET in parallel lines and let dry at room temperature under continuous air flow for 60 minutes. The enzyme mixture is then deposited directly on top the dried primer mixture and let dry for another 60 minutes at room temperature under continuous air flow. PET with deposited dried reagents is then cut into 1 x 1 cm segments which corresponds to one 25 µL reaction.

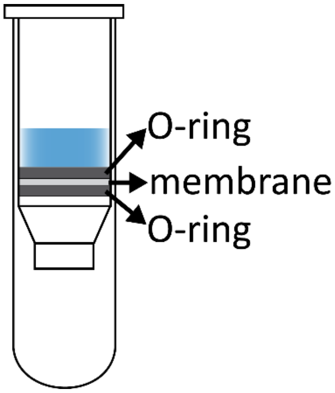

Figure S2. Vertical flow filtration setup. Membrane of interest was placed between two O-rings (after removing commercial filter in Qiagen spin column) and placed into spin column before solution was added.

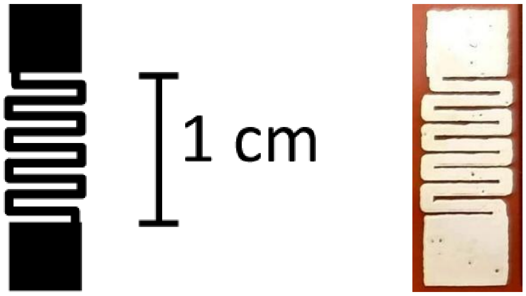

Figure S3. Resistive heating elements. Design and image of the resistive heating element after the printing and curing process.

Table S4. Components and cost of the consumable components of microRAAD.

	**Component**	**Manufacturer**	**Cost/Device**
µPAD	Glass fiber	Millipore	$ 0.02
	MF1 blood separator	GE Life Sciences	<$0.01
	0.22 µm polyethersulfone (PES)	Millipore	$ 0.03
	Wax valve strips	Whatman & Xerox	<$ 0.01
	Cellulose L	Whatman	<$ 0.01
	polyethylene terephthalate (PET)	Apollo	<$ 0.01
	LFIA	USTAR	$ 1.80
	Laminate	Swingline SelfSeal	$ 0.05
	polystyrene gasket	Lohmann Precision Die Cutting	$ 0.01
	Double-sided adhesive	Silhouette	<$ 0.01
		Subtotal	***<$ 1.96***

RT-LAMP Reagents	Isothermal Buffer II	New England Biolabs	$ 0.03
	dNTPs	Agilent Technologies	$ 0.05
	Betaine	Millipore Sigma	$ 0.03
	Primers	Integrated DNA Technologies	$ 0.01
	Bst 3.0 Polymerase	New England Biolabs	$ 0.14
	Sucrose, Glycerol, TritonX-100, green dye, DEPC H2O	Various	$ 0.01
		Subtotal	***$ 0.27***
		**Consumable Total**	**$ 2.23**

Table S5. Components and cost of the reusable components of microRAAD.

	**Component**	**Manufacturer**	**Cost/Device**
Resistive heating elements	nanosilver ink	Novacentrix	$ 0.03
	Kapton substrate		$ 0.45
		Subtotal	***$ 0.48***
Temperature Control Circuit	ATmega328P	Microchip Technology	$ 2.14
	Pogo Pins	Mill-Max Manufacturing Corp.	$ 3.96
	MLX90614	Melexis Technologies NV	$ 44.34
	AP3429	Diodes Incorporated	$ 0.42
	Micro USB Female	Amphenol FCI	$ 0.46
	Green LED	Lite-On Inc	$ 0.27
	DAC6311	Texas Instruments	$ 5.22
	N-MOSFET	Infineon Technologies	$ 1.77
	2.2µH Inductor	Bourns Inc.	$ 0.24
	6-pin Female Headers, Right Angle	Sullins Connector Solutions	$ 0.66
	8MHz Crystal	EPSON	$ 0.83
	Tiny Rectangular Button	C&K Components	$ 0.56
	22µF capacitor	Samsung Electro-Mechanics America, Inc.	$ 1.38
	10µF capacitor	Samsung Electro-Mechanics	$ 1.26
	100n capacitor	Samsung Electro-Mechanics America, Inc.	$ 0.70
	22p capacitor	Samsung Electro-Mechanics America, Inc.	$ 0.30
	10k resistor	Stackpole Electronics Inc.	$ 0.60
	0.1Ohm resistor	Panasonic Electronic Components	$ 1.35
	1k resistor	Bourns Inc.	$ 0.40
	300k resistor	Yageo	$ 0.10
	95.3k resistor	Yageo	$ 0.10
	Printed circuit board (PCB)		$ 0.50
		Subtotal	***$67.56***
Plastic housing	plastic housing	Stratasys	$ 1.89
	acrylic lid	Shape Products	$ 0.15
		Subtotal	***$ 2.04***
		**Reusable Component Total**	**$ 70.08**

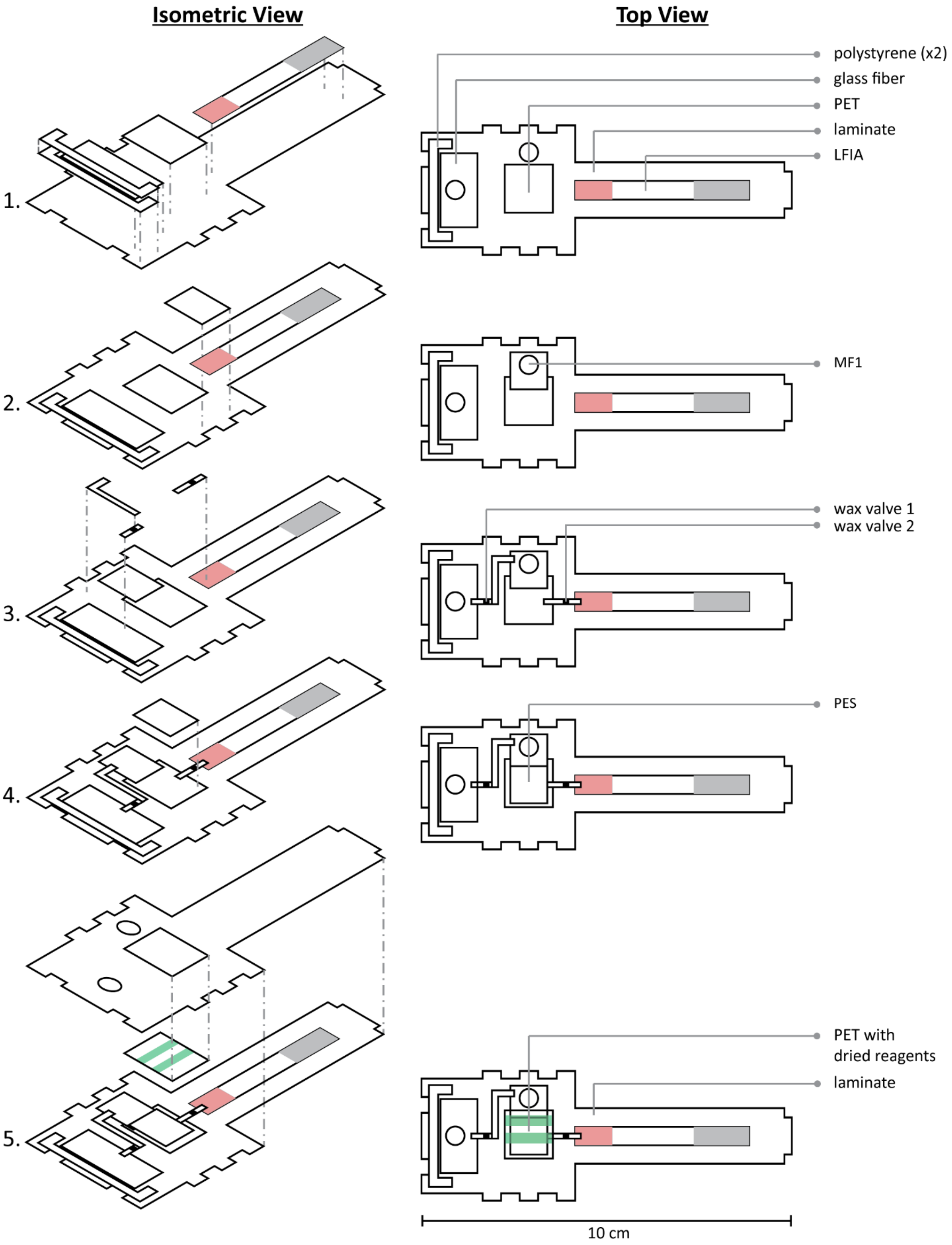

Figure S4. Assembly of µPAD. PES was sandwiched with squares of PET to prevent the laminate from inhibiting amplification.

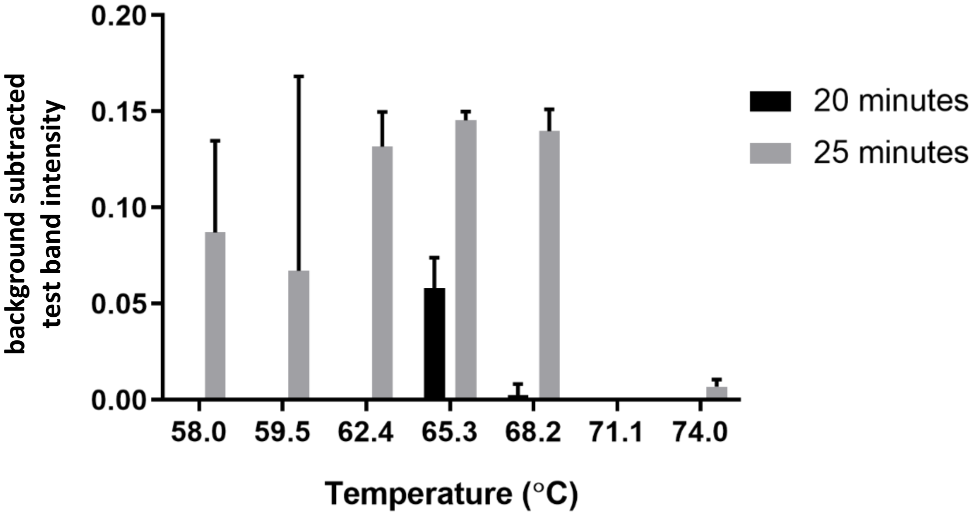

Figure S5. RT-LAMP assay efficiency at various temperatures. Amplification products were analyzed by LFIA after 20 and 25 minutes of heating at temperatures ranging from 58 - 74 ºC. When heated for 20 minutes, only amplicons heated at 65 ºC were detectable on LFIAs, indicating HIV RT-LAMP is optimally efficient at 65 ºC. n=2

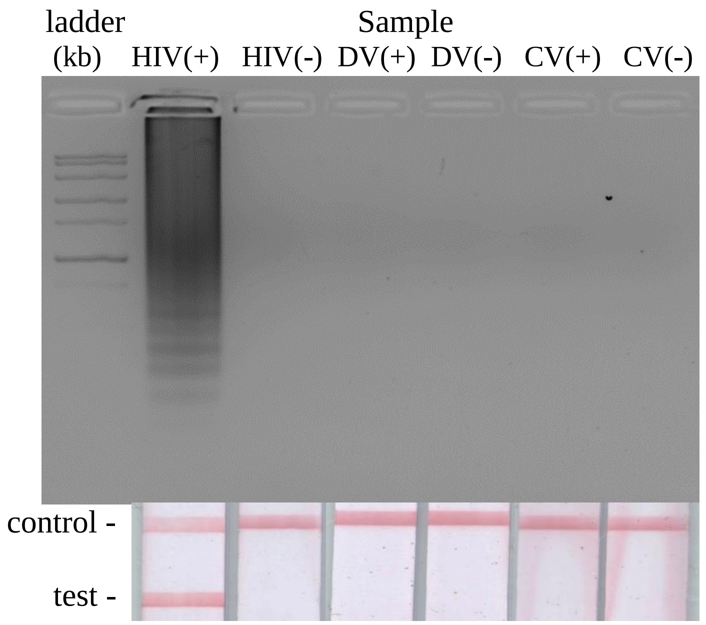

Figure S6. Specificity of HIV LAMP primers. Using RNA from Dengue Type 1 Virus (DV) and Chikungunya S-27 Virus (CV) at a concentration of 10^5 RNA copies/reaction, the HIV RT-LAMP assay was performed for 60 minutes at 65ºC. The only sample that is positive by both gel electrophoresis and LFIA is the HIV(+) sample.

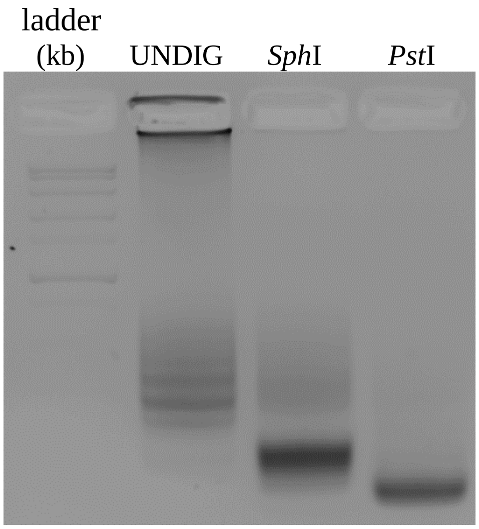

Figure S7. Restriction digest of HIV RT-LAMP amplicons. Restriction enzymes *Sph*I and *Pst*I were used to cut the RT-LAMP product, the amplified *gag* gene. Digested products were compared to the undigested product (UNDIG) using gel electrophoresis.

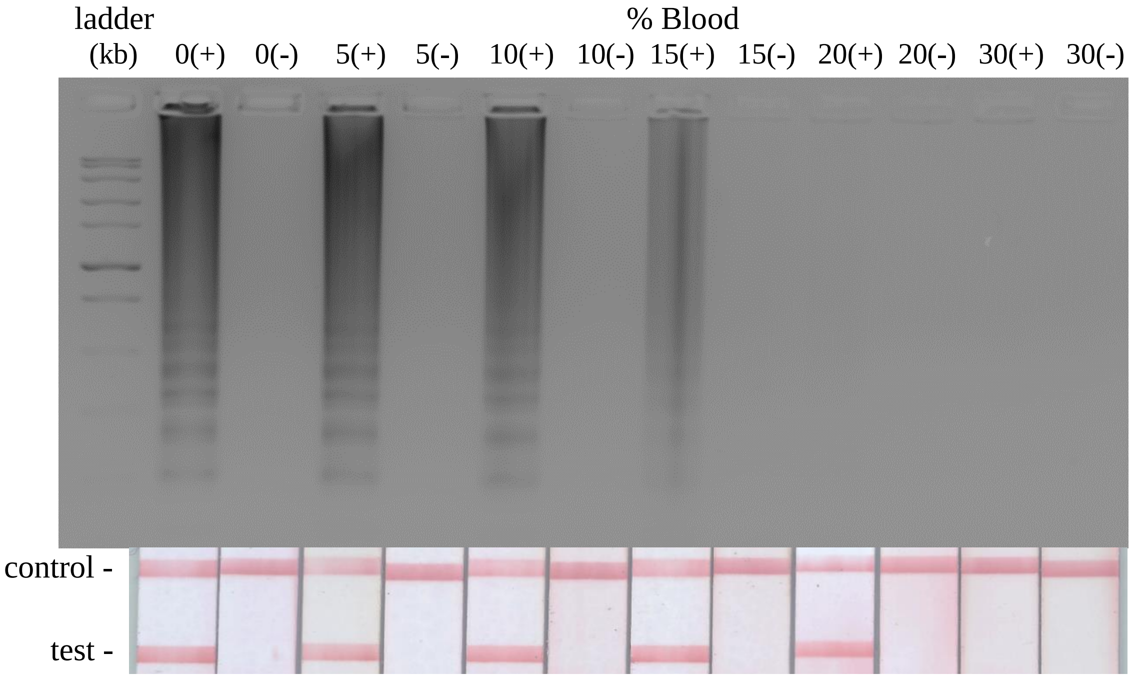

Figure S8. HIV RT-LAMP in human whole blood. HIV-1 virus at a concentration of 10^5 virus copies/reaction was spiked into varying concentrations of blood (0-30%). Gel electrophoresis indicates that the assay tolerance for whole blood is 15% while LFIA demonstrates a tolerance of 20%.

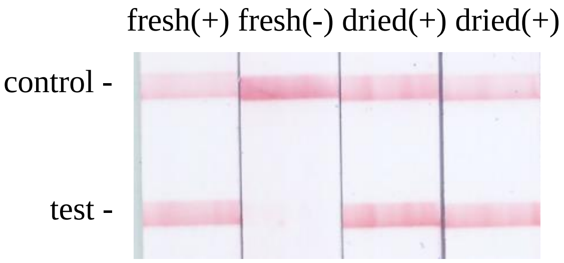

Figure S9. Testing 5-month dried RT-LAMP reagents. Dried reagents were rehydrated with 10^6 virus copies/reaction and rehydrating mixture and amplified for 60 minutes alongside freshly prepared controls.

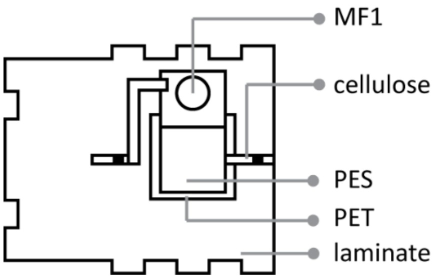

Figure S10. Schematic of MF1/PES assembly for studies verifying red blood cell and virus separation.

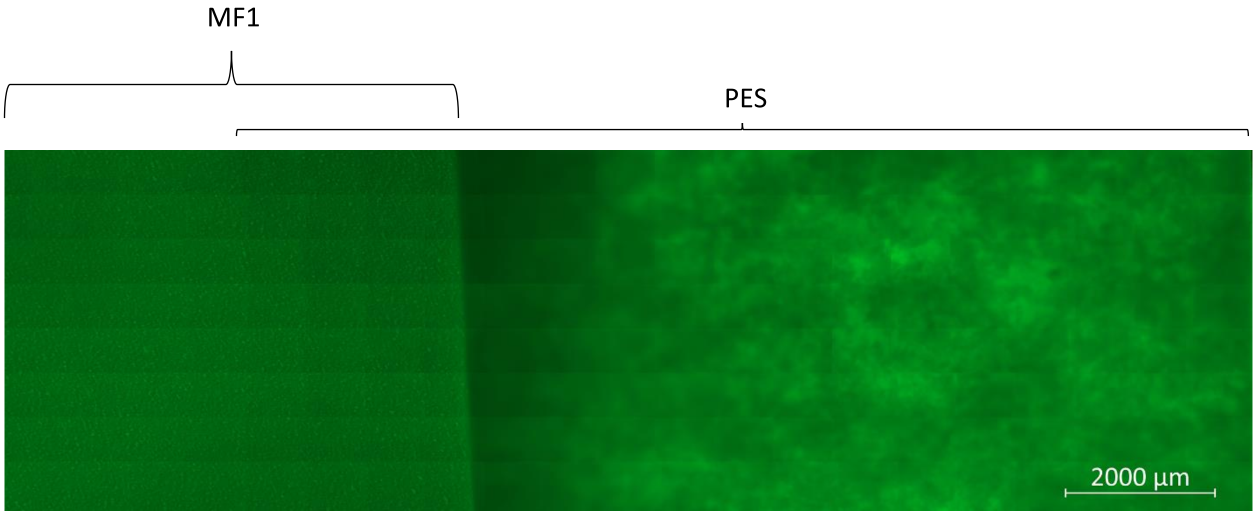

Figure S11. Tiled fluorescent images of 100 nm particles trapped in MF1 and PES of the MF1/PES assembly (Figure S10).

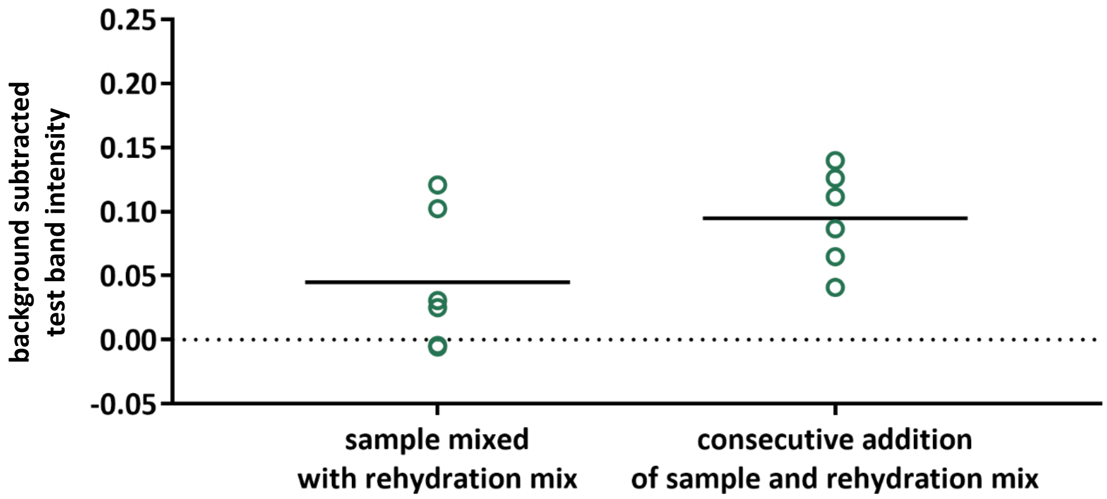

Figure S12. LFIA test band intensity of HIV RT-LAMP products after virus and blood cell separation in MF1. 10^5 copies of HIV virus were diluted in blood and applied to the MF1 of an MF1/PES assembly (Figure S10). Rehydrating mixture was either mixed with the virus in blood and applied simultaneously or applied after the virus in blood (consecutive addition). The PES was removed from the assembly and amplified in RT-LAMP master mix. When the spiked blood was mixed with the rehydrating buffer and applied simultaneously to the MF1 (left), more red blood cells migrated to the PES and inhibited the RT-LAMP assay. n=6.

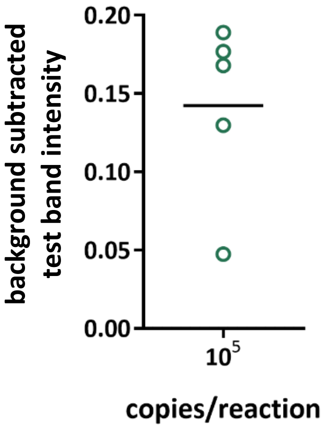

Figure S13. Test band intensity of LFIAs when HIV virus was diluted in water and loaded with fresh RT-LAMP reagents into the microRAAD. n=5

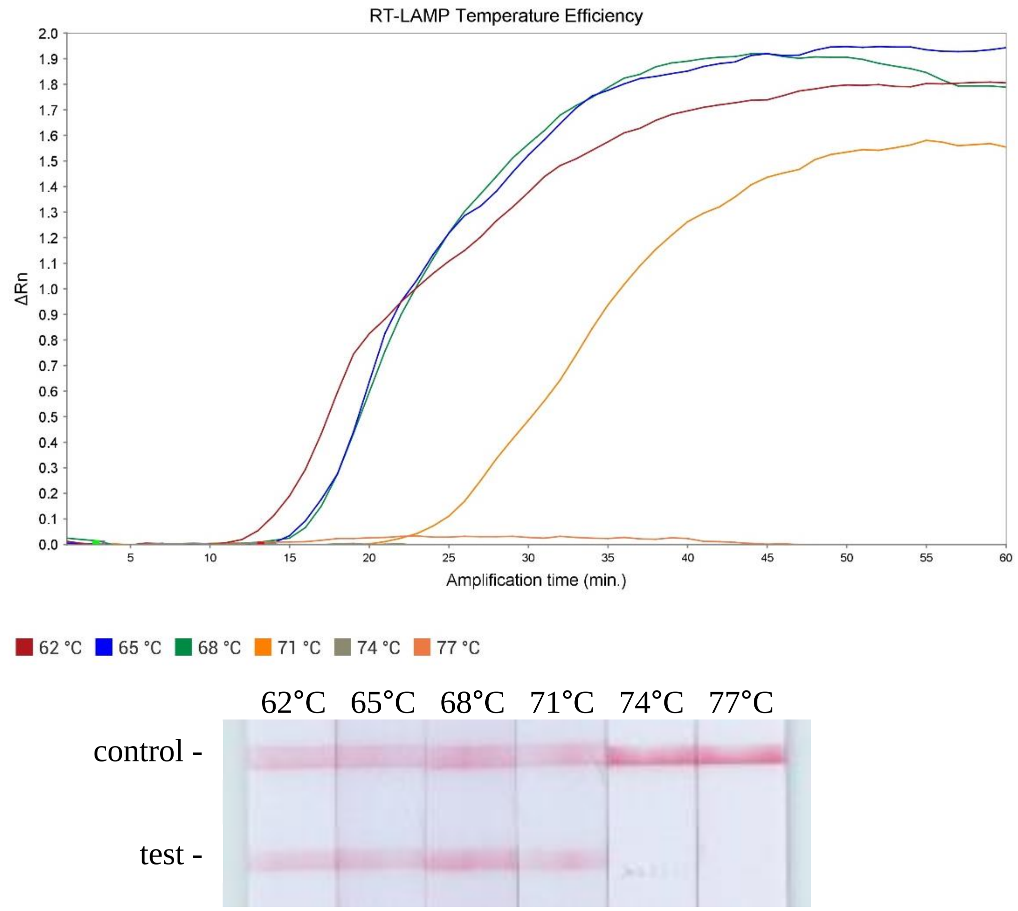

Figure S14. RT-LAMP assay efficiency at various temperatures. Amplicons were analyzed in real-time by fluorescence measurements and LFIA after 60 minutes of heating at temperatures ranging from 62ºC - 77ºC. When the 10^2 RNA copies/reaction template was heated between 62ºC and 68ºC, there was minimal change in time to amplification (demonstrated in real time plot). When heated at 71ºC, amplification was delayed and when heated above 74ºC, no amplification occurred. This result aligns with New England Biolabs' product specification that reverse transcriptase is inactive above 72ºC. Together, this indicates that this RT-LAMP assay for HIV is optimal between 62ºC and 68ºC. n=1
